# Comparison of the tumor microenvironments of squamous cell carcinoma at different anatomical locations within the upper aerodigestive tract in relation to response to ICI therapy

**DOI:** 10.1002/cti2.1363

**Published:** 2022-01-10

**Authors:** Maurice van Duijvenvoorde, Sarah Derks, Idris Bahce, C René Leemans, Rieneke van de Ven, Marieke F Fransen

**Affiliations:** ^1^ Department of Pulmonary Diseases Amsterdam UMC, location VUmc Cancer Center Amsterdam and Amsterdam Institute for Infection and Immunity Amsterdam The Netherlands; ^2^ Department of Otolaryngology Head and Neck Surgery Amsterdam UMC, location VUmc Cancer Center Amsterdam and Amsterdam Institute for Infection and Immunity Amsterdam The Netherlands; ^3^ Department of Medical Oncology Amsterdam UMC, location VUmc Cancer Center Amsterdam and Amsterdam Institute for Infection and Immunity Amsterdam The Netherlands; ^4^ Oncode Institute Utrecht The Netherlands

**Keywords:** esophagus, head and neck, lung, squamous cell carcinoma, tumor microenvironment, ICI‐response

## Abstract

Immunotherapy with immune checkpoint inhibitors (ICI) has improved treatment outcomes in many cancer types and has focused attention on cancer immunity and the role of the tumor microenvironment (TME). Studies into efficacy of immunotherapy and TME are generally restricted to tumors in one anatomical location, while the histological type may have substantial influence on the contexture of the TME, perhaps more so than anatomical location, and subsequently to the response to immunotherapy. This review aims to focus on the TME in ICI‐treated tumors of the same histological type, namely carcinogen‐induced squamous cell carcinoma developing within the aerodigestive tract, at three locations, i.e. head and neck (HNSCC), esophagus (ESCC) and lung (LUSC).

## Graphical abstract

In this review, we focus on the tumor microenvironment in immune checkpoint inhibitor (ICI)–treated tumors of the same histological type, namely carcinogen‐induced squamous cell carcinoma developing within the aerodigestive tract, at three locations, i.e. head and neck, esophageal, and lung squamous cell carcinoma (SCC). We conclude, based on the available literature, that there are many similarities among SCC arising in distinct anatomical locations. However, the differences observed in immune landscapes, in the future, ICI treatments (and other immunotherapeutics) are probably best chosen based on immune cell infiltrate.

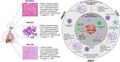

## Introduction

Squamous cell carcinoma (SCC) is an epithelial malignancy that arises on the surface of the skin and in the linings of the aerodigestive tract and anogenital region. Incidence rates of these tumors are rising due to major risk factors such as ultraviolet light, smoking, excessive alcohol consumption and human papillomavirus (HPV) infection.^1^ Treatment of SCC is comparable across the different anatomical sites mentioned above. Standard treatment options include local therapies such as surgery or (chemo‐)radiotherapy in early and advanced, but curable disease, while in the metastatic stage the mainstay is systemic therapy, which is typically a platinum‐based chemotherapy regimen.

Immunotherapy, based on immune checkpoint inhibitors (ICI), is a recent addition to the treatment options for cancer patients, including those diagnosed with SCC. Immunotherapy aims to (re)activate an anti‐tumor immune response, which can be suppressed by factors present within the tumor microenvironment (TME).^2^ Despite the improvement in survival outcomes in clinical studies observed with ICI treatment in multiple types of cancer, only a minority of patients benefit from a durable response to these therapies. In order to increase their efficacy, more research is needed to unravel the mechanisms of action and resistance and to understand the factors within the TME across multiple tumor types.^2^

To date, the use of ICI in SCC, either in the clinic or in research, is mostly in context of each anatomical location or organ. This approach may be reconsidered, as shown in a recent study, indicating that carcinomas at different anatomical sites, but from a same histological subtype, share more similarities than those tumors from different histological subtypes within the same anatomical site.^3^

In this review, we compare the results from studies describing the immune landscapes of SCC in the head and neck region, the esophagus and the lung.^4^, ^5^ Furthermore, the efficacy of ICI is discussed and compared between these tumors. Finally, these reported efficacies are discussed in relation to observed differences in the SCC‐immune landscapes.

## The immune landscape of head and neck squamous cell carcinoma

Head and neck squamous cell carcinoma (HNSCC) is an epithelial malignancy with an average 5‐year survival of 40–50% in the advanced stages.^5^ HNSCC arises mostly in the linings of the oral cavity, pharynx (oropharynx and hypopharynx) and larynx.^6^ Classical risk factors for this disease are smoking and excessive alcohol consumption; however, recent developments showed that HPV infection has an increasing role in the development of these tumors. HPV‐positive (HPV+) HNSCC develops mainly in the oropharynx, and is generally associated with improved outcome.^7^ A new understanding of the genomic landscape of HNSCC has emerged, pointing out crucial differences between HPV+ and HPV‐unrelated (HPV−) tumors, while in both tumors a high mutational burden is observed, distinct oncogenic pathways are involved.^8^ Since HPV infection drastically alters the TME of tumors and is rarely involved in LUSC and ESSC, we will focus our review on HPV‐ HNSCC, specifically.

Previously, research into the immune landscape of HNSCC suggested an immunosuppressive tumor milieu and a minor role for T cells with reduced proliferation, signaling abnormalities and increased apoptotic signals.^9^, ^10^, ^11^, ^12^ Surprisingly, recent studies point out more contrasting results, ranging from highly immune infiltrated or ‘hot’ tumors to tumors lacking immune infiltrate or ‘cold’ tumors, at different anatomical locations within the head and neck area, which suggests a highly heterogeneous immune landscape.^13^, ^14^, ^15^ Mandal *et al*.^13^ found that HNSCC is among the highest immune infiltrated cancer types in general. This was observed at all different anatomical sites within HNSCC. The presence of tumor‐infiltrating lymphocytes (TILs) in HNSCC generally has been correlated with improved prognosis and outcomes.^16^ Compared to other tumor types, HNSCC in general had the highest infiltration of Tregs and CD56^dim^ NK cells. CD56^+^ NK cells were associated with increased survival in tumor samples^17^. High levels of Tregs are found in HNSCC along with high levels of CD8^+^ T cells. Surprisingly, Tregs are often associated with good prognosis and outcome in HNSCC.^13^, ^18^, ^19^ In contrast, tumors in patients with a history of heavy smoking, were associated with low levels of immune infiltrate, suggesting an immune ‘cold’ TME.^13^ These studies suggest that there are differences in the immune infiltrate in distinct anatomical locations within HNSCC, but most of these differences can be attributed to the HPV status, with the HPV‐unrelated, smoking and alcohol–induced HNSCC in general displaying an immune cold or excluded TME with low levels of T‐cell infiltration.^20^

HNSCC TMEs are rich in non–T‐cell immune cell subsets. Chronic inflammation is induced in many HNSCC through the expression of pro‐inflammatory and pro‐angiogenic cytokines, which leads to the recruitment of myeloid derived suppressor cells (MDSCs) and tumor‐associated macrophages (TAMs).^11^, ^21^ Hanna *et al*. observed high levels of granulocytes and monocytes in SCC of the oral cavity and larynx and suggested a prominent role of MSDCs in these tumors.^14^ It has long been established that the presence of MDSCs is associated with poor prognosis and outcome,^22^, ^23^ regulated by the recruitment of CD34^+^ immune cells due to secretion of GM‐CSF by HNSCC cells.

## The immune landscape of esophageal squamous cell carcinoma

Worldwide, squamous cell carcinoma is the most prevalent histological subtype of esophageal cancer; however, in Western populations adenocarcinomas are much more dominant. Given the difference in etiological drivers and molecular background, squamous cell carcinomas and adenocarcinomas of the esophagus are considered distinct entities. Squamous cell carcinomas of the esophagus show more similarity with head and neck cancers than esophagogastric adenocarcinomas. ESCC are one of the most aggressive cancers with a poor prognosis, especially when these cancers are detected at an advanced stage which is associated with a 5‐year survival rate of < 5%. Smoking and alcohol consumption are also major risk factors for ESCC.

In recent years, accumulating data have shown that also the tumor immune microenvironment of ESCCs is dominated by exhausted T cells, and suppressive cell populations such as Tregs, MDSCs and M2‐type, suppressive macrophages.^24^ This is a remarkable finding as ESCCs have a high mutational load which is often associated with an activated antitumor‐immune response.^25^ The suppressed immune microenvironment in ESCC can potentially be explained by the chronically inflamed environment in which these cancers develop. Risk factors smoking and alcohol intake have been shown to induce a chronic inflammatory state and the production of reactive oxygen species (ROS), which subsequently induces DNA damage and activation of multiple cancer‐associated pathways such as the nuclear factor‐κB (NF‐κB) pathway in the esophagus.^26^ Furthermore, chronic inflammation stimulates expression of cytokines such as IL‐6 and TNF‐α which also have anti‐apoptotic and immune suppressive properties.^26^ IL‐6 for instance inhibits the maturation of DC and promotes alternative activation (M2‐type) of macrophages and thereby compromises the priming of tumor‐specific T cells. Thereby the chronic inflammatory state is a central driver of the complex pro‐tumoral and anti‐inflammatory immune environment that is typical for ESCCs.

There have been multiple studies to acquire a detailed understanding of the key players of the suppressed immune microenvironment of ESCCs. One of the most systematic evaluations performed single‐cell RNA sequencing (scRNAseq) of seven ESCCs and identified that some ESCCs actually have high numbers of tumor‐infiltrating T cells but that the majority of the proliferating immune cells are exhausted CD4/8 T cells and NK cells.^24^ The tumor‐infiltrating, exhausted NK cells were found to express the checkpoint molecules NGK2A and CD49B.^24^ A very recent scRNAseq study, performed on 60 ESCC and four healthy control tissues, confirmed the high levels of exhausted T cells within ESCC, especially in more advanced‐stage disease, as well as increased Treg frequencies as opposed to identified naïve, memory or effector T‐cell subsets.^27^ These data further indicated that the exhausted phenotype T cells, most likely reflected tumor‐reactive T cells, given their high expression of CD39 and CD103 and low levels of KLRG1.^28^

Furthermore, the composition of the immune microenvironment differs greatly between ESCCs. Using mRNA sequencing data, Lin *et al*. divided 81 ESCCs into a group with a high and a group with a low immune score.^3^ In this series, immune scores were not associated with tumor stage, but did show an association with tumor grade, suggesting that the immune composition impacts tumor cell differentiation. Interestingly, an association between inflammatory programs and muscle metabolisms was identified, which needs further evaluation.^8^ Also, in these series, enrichment of CD4 memory T cells, M1 Macrophages and M2 Macrophages signatures was associated with worst outcome, while B‐cell enrichment was associated with an improved outcome^8^. These results are in agreement with another study that used RNA expression data from The Cancer Genome Atlas to analyze the ESCC immune infiltrate and showed that B‐cell enrichment was associated with an improved outcome^9^. Within this last study, B cells were shown to cluster in tertiary lymphoid structures, which are often associated with a favorable outcome.

Macrophages are most often associated with a poor outcome. However, macrophages can be pro‐inflammatory or anti‐inflammatory. Single‐cell mRNA expression analyses in ESCC revealed that M1 and M2 macrophage‐associated gene patterns often coexist in the same cells, indicating that tumor‐associated macrophages are more complex than the classical M1/M2 model.^24^ The effect of targeting macrophages in these tumors is therefore not immediately clear.^24^ Zhang *et al*. using scRNAseq data from 60 ESCC tumors showed that from the myeloid cells identified in the ESCC TME, tolerogenic dendritic cells (tDCs), expressing high levels of PD‐L1 and PD‐L2, showed the most ligand/receptor interactions with CD8 T‐cell subsets compared to other DC subtypes present. They additionally performed *in vitro* stimulation assays of isolated tDCs with autologous CD8^+^ T cells and showed effective suppression of T‐cell proliferation, which was dependent on PD‐1/PD‐L1 interaction.^27^

## The immune landscape of squamous non‐small cell lung cancer

Lung cancer is the leading cause of cancer‐related death worldwide with a 5‐year survival rate of approximately 15%.^5^ Lung cancer consists of distinct histologic subtypes, with small‐cell lung cancer accounting for 15% of the cases and non‐small cell lung cancer accounting for the remaining 85%. In turn, NSCLC is divided into adenocarcinoma (40%), SCC (25–30%) and large cell carcinomas (5–10%)^29^. A major risk factor for the development of NSCLC is smoking, leading to high mutational burden observed in these tumors.^30^ The different histologic subtypes within NSCLC each are associated with a different mutational profile; however, heterogeneity is observed within the genomic landscape of squamous cell lung cancer (LUSC) as well.^29^, ^30^

LUSC was proposed to be a highly immune infiltrated tumor by Kargl *et al*.^31^ By using flow cytometry, they identified that over 50% of the tumor area consists of CD45^+^ immune cells and CD45^+^ cells were approximately three times more abundant in tumor tissue versus healthy tissue, including elevated frequencies of B‐cell and T‐cell subsets. The CD4^+^ T‐cell compartment in LUSC was found to be composed of increased levels of Tregs and reduced levels of Th1 and Th17 T cells in comparison to healthy lung tissue. Higher CD8^+^ T‐cell infiltrate was found in LUSC tumor tissue compared to adjacent healthy lung tissue and showed expression of the activation marker CD69, indicating an activated CD8 phenotype. Notably, CD8 memory T cells marked as CD8_EMRA_ were significantly reduced in LUSC, as well as NK‐cell levels in NSCLC in general. Furthermore, IFN‐γ production by CD8^+^ T cells in LUSC was comparable to normal lung tissue ^31^. These studies give a valuable indication of composition of the immune landscape in LUSC.

The prognostic relevance of TILs has been investigated intensively through recent years (recently reviewed by Bremnes *et al*.).^32^ In general, the infiltration of TILs into the tumor area is associated with positive impact on prognosis and ICI treatment outcomes.^32^ As compared to TILs in the tumor compartment, TILs in the tumor stroma are a stronger predictor of PFS and OS in ICI treated patients.^33^ As mentioned before, reduced levels of NK cells are generally observed in NSCLC compared to healthy lung tissue.^31^ Nonetheless, NK cells are important mediators of the antitumor response via direct and indirect cytotoxic mechanisms.^34^ Kargl *et al*. showed that cells of the myeloid lineage were the most frequent cell type found in LUSC, representing 50% of CD45^+^ cells.^31^ In this study, neutrophilic granulocytes accounted for 20% of the total CD45^+^ cell population and showed a negative interaction with CD8^+^ T cells. Approximately 10% of the CD45^+^ cells were composed of monocytes and 60% of these monocytes were HLA‐DR^LO^, so‐called monocytic‐MDSC.

These high levels of myeloid lineage cells present in LUSC have implications on disease prognosis and ICI outcomes as well. A high tumor‐associated neutrophil density was previously identified as an independent positive prognostic factor for disease‐specific survival in LUSC.^35^ Moreover, the presence of CD11b^+^/CD14⁻/CD15^+^/CD33^+^ granulocytic‐MDSCs and CD14^+^/S100A9^+^ monocytic‐MDSCs, expressing L‐arginase and nitric oxide synthase, in the tumor area, resulted in suppression of CD8^+^ T cells and correlated with reduced survival.^36^, ^37^

Tertiary lymphoid structures (TLS) are cellular aggregates which are organized as a multicellular lymphoid organ.^38^, ^39^ Many NSCLC patients present with these structures, which are associated with improved prognosis and ICI treatment outcome.^40^ TLS contain mature dendritic cells, mostly found in a T‐cell zone close to a B‐cell follicle, resembling a lymph node structure, generating a T‐cell mediated adaptive immune response against the tumor.^40^ Germain *et al*. found that TLS in NSCLC exhibit B‐cell–related immune responses and that a high density of follicular B cells was associated with improved survival in early‐ and advanced‐stage disease.^41^ Lizotte *et al*. observed that 20 of the 22 analyzed tumors contained TLS, which contained elevated levels of immune‐suppressing, IL‐10 secreting, B‐regulatory lymphocytes compared to healthy lung (approximately 11% of the total B‐cell population).^42^ Furthermore, high mature dendritic‐cell density within the TLS was associated with high T‐cell infiltration and gene signatures related to Th‐1 and cytotoxic T‐cell phenotype. High levels of mature dendritic cells in TLS correlated with improved survival as well.^43^ These studies indicate the importance of TLS in the anti‐tumor response in LUSC.

## The distribution of immune landscape differs between SCC of the head and neck, esophagus and lung

Previous reports suggested that tumors could best be re‐categorized by their biology, which would lead to treatments based on molecular characteristics instead of general treatment options per anatomical location.^3^ To investigate whether treating patients with ICI based on molecular characteristics instead of anatomical location could lead to improvements in therapy efficacy, the immune landscapes of similar malignancies arising in distinct anatomical locations should be compared.

A major risk factor which is shared between HNSCC, ESCC and LUSC is smoking and differences in the immune landscapes of smokers and non‐smokers are recognizable. Smokers generally have a higher mutational burden with specific mutational profiles than non‐smokers, resulting in more neo‐antigens that can potentially be recognized by the immune system as non‐self. In LUSC, a higher mutational burden due to smoking has been associated with increased immune cell infiltrate and inflammation.^44^ In HNSCC, however, a smoking‐related high mutational load was associated with reduced immune cell infiltrate, strong immunosuppressive effects, and poor survival, indicating a suppressed anti‐tumor response.^44^ Also, in ESCC, a high mutational load is not clearly associated with immune cell activation. These observations demonstrate that a high mutational burden due to smoking has different effects in SCC depending on the distinct anatomical location.

Additionally, TLS are found in both HNSCC and LUSC; however, the occurrence of these structures is far higher in LUSC (> 95%) than HNSCC (21%).^45^, ^46^ For ESCC, a clear percentage could not be retrieved from current literature. In HNSCC, a TLS profile, with high expression of genes related to T follicular helper cells (Tfh), was linked to improved survival, but this was mostly apparent in HPV+ tumors.^47^ A recent study in early‐stage (cT1N0) oral tongue SCC, which are predominantly HPV−, showed TLS to be present in 76.3% of the cases and that presence of TLS was related to a favorable prognosis^20^, ^48^. This again clearly exemplifies the importance of dissecting the immune composition at the separate anatomical sites within HNSCC, also in view of selecting those patients with tumors in a HNSCC subsite that might be more responsive to ICI therapy. In ESCC, interaction between Tfh and germinal center B cells, derived from scRNAseq data, clearly indicated the presence of a TLS‐rich environment in a subset of ESCC.^27^

A large study, investigating over 10 000 tumors across 33 types of cancer, using data from the TCGA, found six immune subtypes (IS): inflammatory, IFN‐γ dominant, wound healing, lymphocyte depleted, immunologically quiet and TGF‐β dominant.^49^ Although these six IS could be identified in nearly all malignancies, 90–95% of the SCC belonged to either the wound healing or IFN‐γ dominant IS. In turn, Li *et al*. identified IS that were more specific to SCC of distinct anatomical locations, also using gene expression profiles of the TCGA.^50^ In this study again six IS were described based on seven gene expression profiles, shown in Table 1. Differences could be observed in immune subtypes between HNSCC and LUSC; LUSC was enriched in IS1 (intermediate immune infiltrate, high M2‐macrophage polarization and biased to humoral immunity) and IS5 (high inflammation, reactive stroma and TGF‐β, indicating immune hot/suppressed TME). In HPV‐unrelated HNSCC, also IS1 and IS5 were most prevalent, but in addition there was a large proportion of tumor (19%) with an IS2 signature (Intermediate immune cell infiltrate, immune suppressing phenotype with high TGF‐β signature; high IFN‐γ signature; high M1‐macrophage signature) (Table 1). ESCC had a similar contribution of IS1 and IS2 compared to the HNSCC, but the most prominent signature observed in these tumors (36%) was IS3 (immune cold; low expression of genes related to inflammation, reactive stroma, T cells and IFN‐γ; high mutational burden; high TGF‐β signature).

**Table 1:** Immune subtypes observed in SCC of the cervix, lung, esophagus and head and neck based on 7 immune gene signatures observed in a study performed by Li *et al*.^50^

	Angiogenesis	Inflammation	Reactive stroma	T‐cell specific	IFN‐γ related	TGF‐β	Differentiation	NSCLC	HNSCC	ESCC	Molecular and cellular characteristics
IS1	High	Intermediate	High	Intermediate	Low	High	Low	41%	30%	29%	Intermediate immune cell infiltrate, towards immune suppressing phenotype (high TGF‐β signature); high angiogenesis, reactive stroma signature and M2‐macrophage signatures; biased to humoral immunity, with high levels of naïve B‐cells and plasma cells; high mutational burden
IS2	Intermediate	Intermediate	Intermediate	Intermediate	High	High	High	1%	19%	17%	Intermediate immune cell infiltrate, towards immune suppressing phenotype (high TGF‐β signature); highest IFN‐γ signature; high M1‐macrophage signature
IS3	Intermediate	Low	low	Low	Low	High	Low	14%	14%	36%	Immune cold; low expression of genes related to inflammation, reactive stroma, T‐cells and IFN‐γ; high mutational burden; high TGF‐β signature
IS4	Intermediate	High	Intermediate	High	High	Low	Low	5%	8%	5%	Immune hot with existing anti‐tumor response; high immune cell infiltrate with enrichments in CD4+ memory T‐cells, follicular T‐helper cells, CD8+ T‐cells and NK‐cells; elevated levels of M1‐M2 macrophage ratio and IFN‐γ; suppressed TGF‐β signature
IS5	High	High	High	High	Intermediate	High	Low	35%	21%	6%	Immune hot although suppressed; highest angiogenesis, inflammation, reactive stroma and TGF‐β signature with approximately 40% M2‐macrophages; high T‐cell and intermediate IFN‐γ signature, likely in suppressed state
IS6	Low	Low	Low	Intermediate	Intermediate	Low	Low	5%	7%	6%	Immune cold; Low expression of genes related to angiogenesis, inflammation and reactive stroma, with lowest TGF‐β signature; intermediate T‐cell and IFN‐γ gene signature including activated CD8+ T‐cells and NK‐cells

This study gives important insight into the differences between the immune landscapes of SCC. For example, IS1 and IS3 were associated with a high mutational burden, which may be related to smoking status. Moreover, IS1 was observed in 41% of LUSC and was biased to humoral immunity, containing high levels of naïve B cells and plasma cells. This may be associated with the observation that TLS are more abundant in these tumors compared to HNSCC, since humoral immunity forms an important association to TLS.^41^, ^46^ In general, the ESCC seem to include the most immune cold tumors of the three SCC sites.

## The difference between immune checkpoint expression in SCC of the head and neck, esophagus and lung

An important factor in suppressing the anti‐tumor response in HNSCC, ESCC and LUSC is elevated expression of immune checkpoints (IC). Expression levels can be assessed either as tumor proportion score (TPS), looking at PD‐L1 membrane expression on tumor cells only, or the combined positive score (CPS), which takes into account partial or complete membrane PD‐L1 expression on the tumor cells as well as PD‐L1 expression (membranous and cytoplasmic) on infiltrating lymphoid and myeloid cells. In HNSCC, expression levels of PD‐L1^51^ is reported to be highly variable, ranging from 18% to even up to 87% TPS in all anatomical locations. Tumor PD‐L1 expression heterogeneity may lead to a misrepresentation as biopsies are taken from a limited number of sites. Taking six biopsies from the same tumor excision specimen (*n* = 33 tumors in total), Rasmussen *et al*. showed that the concordance between the biopsies for the TPS was 36% and for the CPS 52% when the cut‐off was set at 1%.^52^ For a cut‐off of > 50%, the concordance was 77% for TPS and 55% for CPS. Moreover, the negative predictive value (NPV) of a single negative biopsy at a 1% cut‐off was 38.9% and 0% for TPS and CPS, respectively. For a cut‐off of > 50%, the NPV was 79.9% for TPS and 62.8% for CPS. These data can contribute to the large variance in PD‐L1 positivity observed between patients, as well as the observations that tumors scoring PD‐L1 negative based on immunohistochemistry do respond to anti‐PD(L)1 treatment and stress the need for more reliable biomarkers than PD‐L1 expression for anti‐PD‐1 immune checkpoint inhibitor (ICI) therapy.

The same alternating results are observed in LUSC in terms of PD‐L1 expression and prognostic value. A study performed by Pawelczyk *et al*. compared their own findings in terms of PD‐L1 expression with other studies. In most studies, PD‐L1 expression levels were at 50–70%, with a few studies differing by small percentages. To note, most studies that found high PD‐L1 expression were performed in an Asian study population. In studies investigating Western populations, lower levels of PD‐L1 were observed.^53^

Both PD‐L1 and PD‐L2 are common features of ESCCs and are associated with a worst outcome.^5^, ^6^ Also, CTLA‐4, PD‐1, TIM‐3 and LAG‐3 are often upregulated and associated with more aggressive features such as depth of invasion, lymph node involvement and also epithelial to mesenchymal transition (EMT) in ESCC.^7^

Recent advances show that also in HNSCC and LUSC, other ICs like TIM‐3, LAG‐3, OX40, BTLA and TIGIT are upregulated, giving a rationale for testing these therapeutic targets in clinical studies.^39^, ^51^

## The efficacy of immune checkpoint inhibition in squamous cell carcinoma of the head and neck, esophagus and lung

Through recent years, ICI have intensively been investigated for treatment of many types of cancer,^2^ and, since then, many clinical trials have been performed investigating these ICI in HNSCC, ESCC and LUSC which led to the approval of several ICI for the treatment of these malignancies.^51^, ^54^, ^55^, ^56^, ^57^

The first therapeutic advances of ICI in HNSCC, LUSC and ESCC were seen targeting the PD‐1/PD‐L1 axis. Pembrolizumab and nivolumab are humanized monoclonal antibodies that target the PD‐1 receptor present mainly on T cells, blocking the binding of PD‐L1 and the alternative ligand PD‐L2, to the receptor and reversing the inhibitory signal that suppresses T‐cell activity.^58^, ^59^ Pembrolizumab was first approved by the FDA in 2016 for the treatment of recurrent or metastatic HNSCC that had progressed after platinum‐based chemotherapy, following the Phase I KEYNOTE‐012 trial.^60^ In advanced NSCLC, pembrolizumab was granted accelerated approval in 2016 following the KEYNOTE‐001 study for first‐ and second‐line treatment of this disease. Of note, only about 20% of the intention‐to‐treat population included patients with squamous disease.^61^ In both studies, similar response rates (RR) and OS were observed in the second line treatment, suggesting a similar efficacy in HNSCC compared to NSCLC. Subsequently, the efficacy of pembrolizumab was investigated in comparison with standard of care (SOC) in first line therapy.^62^, ^63^ As shown in Tables 2 and 3, the efficacy of first line pembrolizumab vs. SOC was greater in NSCLC than in HNSCC. In the first‐line treatment settings of HNSCC and LUSC, pembrolizumab was investigated in combination with SOC as well.^64^, ^65^ When comparing the efficacy of pembrolizumab in combination with chemotherapy between HNSCC and LUSC, the prolongation of median OS is higher in LUSC than in HNSCC.

**Table 2:** Clinical trials performed in HNSCC for pembrolizumab, nivolumab, durvalumab and atezolizumab

Study name	Phase	Line of treatment	Design	HPV status (positive (%)) (ICI treatment vs. SOC)	PD‐L1 expressing patients – TPS (percentage) (ICI treatment vs. SOC)	PD‐L1 expressing patients – CPS (percentage) (cut‐off)	Median follow‐up (ICI treatment vs. SOC)	Response rate	Median OS (ICI treatment vs. SOC)
KEYNOTE‐012^60^	Phase Ib	Second line or later	Pembrolizumab alone	45 (23%)	123 (65%) TPS cut‐off ≥ 1%	152 (81%) CPS cut‐off ≥ 1	9.0 months	18%	8.0 months
KEYNOTE‐040^62^	Phase III	Second line or later	Pembrolizumab vs. methotrexate/docetaxel/cetuximab	61 (25%) vs. 58 (23%)	64 (26%) vs. 65 (26%) TPS cut‐off ≥ 50%	196 (79%) vs. 191 (77%) CPS cut‐off ≥ 1	7.5 vs. 7.1 months	NA	8.4 vs. 6.9 months (HR 0.80; 95% CI 0.65–0.98; *P* = 0.0161)
KEYNOTE‐048^64^	Phase III	First line	Pembrolizumab alone or with chemotherapy vs. cetuximab with chemotherapy	63 (21%) vs. 67 (22%) ‐ ICI alone vs. SOC 60 (21%) vs. 61 (22%) ‐ ICI with chemotherapy vs. SOC	67 (22%) vs. 66 (22%) ‐ ICI alone vs. SOC 66 (23%) vs. 62 (22%) ‐ ICI with chemotherapy vs. SOC TPS cut‐off ≥ 50%	133 (44%) vs. 122 (41%) ‐ ICI alone vs. SOC 126 (45%) vs. 110 (10%) ‐ ICI with chemotherapy vs. SOC CPS cut‐off ≥ 20	11.5 months ‐ ICI alone 13.0 months ‐ ICI with chemotherapy 10.7 months ‐ SOC	NA	11.5 vs. 10.7 months (HR 0.83; 95% CI 0.70−0.99; *P* = 0.0199) – ICI alone vs. SOC 13.0 vs. 10.7 months (HR 0.77; 95% CI 0.63−0.93; *P* = 0.0034) ‐ ICI with chemotherapy vs. SOC
Checkmate‐141^66^	Phase III	Second line or later	Nivolumab vs. methotrexate/docetaxel/cetuximab	63 (26.2%) vs. 29 (24.0%)	88 (36.7%) vs. 61 (50.4%) TPS cut‐off ≥ 1%	NA	5.1 vs. 5.1 months	13.3% vs. 5.8%	7.5 vs. 5.1 months (HR 0.70; 97.73% CI 0.51–0.96; *P* = 0.01)
HAWK^68^	Phase II	Second line or later	Durvalumab alone	34 (34.4%)	112 (100%) TPS cut off ≥ 25%	NA	6.1 months	16.2%	7.1 months
EAGLE^70^	Phase III	Second line or later	Durvalumab with/without Tremelimumab vs. SoC	30–31 (12.0–12.5%) ‐ In all treatment groups	68–72 (28.3–29.1%) ‐ In all treatment groups, cut‐off ≥ 25%	NA	7.6 months ‐ durvalumab 6.3 months ‐ durvalumab with tremelimumab 7.8 months ‐ SoC	17.9% ‐ durvalumab 18.2% ‐ durvalumab with tremelimumab 17.3% ‐ SoC	7.6 months ‐ durvalumab 6.5 months ‐ durvalumab with Tremelimumab 8.3 months ‐ SoC
PCD4989g^72^	Phase Ia	Second line or later	Atezolizumab alone	13 (46%) 3 patients (11%) unknown	NA	25 (78%) Only on immune cells, cut‐off ≥ 5%	14 months	22%	6.0 months

Factors as HPV‐status and PD‐L1 expression are stated, as well as response rates and median overall survival (NA = not assessed; TPS = tumor proportion score; CPS = combined positive score).

**Table 3:** Clinical trials performed in NSCLC for pembrolizumab, nivolumab, durvalumab and atezolizumab

Study name	Phase	Line of treatment	Design	Histology AC (%) vs. SCC (%)	Smoking status (current/former smoker (%)) (ICI treatment vs. SOC)	PD–L1 expressing patients –– TPS (percentage) (ICI treatment vs. SOC)	PD–L1 expressing patients –– CPS (percentage) (cut‐off)	Median follow–up (ICI treatment vs. SOC)	Response rate	Median OS (ICI treatment vs. SOC)
KEYNOTE‐001^59^	Phase I	First line and second line or later	Pembrolizumab alone	79 (78%) vs. 19 (19%) ‐ first line 367 (82%) vs. 76 (17%) ‐ second line or later	90 (89%) ‐ first line 324 (72%) ‐ second line or later	191 (23.2%) TPS cut‐off ≥ 50% in total population	NA	10.9 months	24.8% ‐ first line 18% ‐ second line or later	16.2 months ‐ first line 9.3 months ‐ second line or later
KEYNOTE‐010^63^	Phase II/III	Second line or later	Pembrolizumab (2 mg kg^−1^ and 10 mg kg^−1^) vs. docetaxel	240 (70%) vs. 76 (22%) ‐ 2 mg kg^−1^ 244 (71%) vs. 80 (23%) ‐ 10 mg kg^−1^ 240 (70%) vs. 66 (19%) ‐ docetaxel	279 (81%) ‐ 2 mg kg^−1^ 285 (82%) ‐ 10 mg kg^−1^ 269 (78%) ‐ docetaxel	All patients TPS cut‐off ≥ 1%	NA	13.1 months – all groups	18% ‐ 2 mg kg^−1^ 18% ‐ 10 mg kg^−1^ 9% ‐ docetaxel	10.4 vs. 8.5 months (HR 0.71; 95% CI 0.58–0.88; *P* = 0.0008) ‐ 2 mg kg^−1^ vs. docetaxel 12.7 vs. 8.5 months (HR 0.61; 95% CI 0.49–0.75; *P* < 0.0001) ‐ 10 mg kg^−1^ vs. docetaxel
KEYNOTE‐407^65^	Phase III	First line	Pembrolizumab with chemotherapy vs. chemotherapy alone	All SCC patients	256 (92.1%) ‐ Pembrolizumab with chemotherapy 262 (93.2%) ‐ chemotherapy alone	176 (63.3%) ‐ Pembrolizumab with chemotherapy 177 (63.0%) ‐ chemotherapy alone TPS cut‐off ≥ 1%	NA	7.8 months ‐ all groups	57.9% ‐ Pembrolizumab with chemotherapy 38.4% ‐ chemotherapy alone	15.9 vs. 11.3 months (HR 0.64; 95% CI 0.49−0.85; *P* = 0.0001)
Checkmate‐017^67^	Phase III	Second line or later	Nivolumab vs. docetaxel	All SCC patients	121 (90%) ‐ Nivolumab 129 (94%) ‐docetaxel	81 (60%) vs. 85 (62%) TPS cut‐off ≥ 1%	NA	Minimum follow–up of 11 months	20% vs. 9.0%	9.2 vs. 6.0 months (HR 0.59; 97.73% CI 0.44–0.79; *P* < 0.001)
ATLINTIC cohort 2^69^	Phase II	Third line or later	Durvalumab alone	210 (79%) vs. 55 (21%)	225 (85%)	149 (56%)	NA	7.0 months	16.4%	10.9 months
MYSTIC^71^	Phase III	First line	Durvalumab with/without tremelimumab vs. SoC	52–53 (31.9–32.5%) ‐ In all treatment groups	138–141 (84.7–81.1%)	162–163 (43.5–43.8%) ‐ In all treatment groups, cut‐off ≥ 25%	NA	30.2 months ‐ For all treatment groups	In patients with TPS ≥ 25–35.6% ‐ Durvalumab 34.4% ‐ durvalumab with tremelimumab 37.7% ‐ SoC	In patients with blood tumor mutational burden (bTMB) ≥ 20 mut/Mb 12.6 months ‐ Durvalumab 21.9 months ‐ durvalumab with tremelimumab 10.0 months ‐ SoC
POPLAR^73^	Phase II	Second line or later	Atezolizumab vs. docetaxel	49 (34%) vs. 48 (34%)	117 (81%) vs. 114 (80%)	48 (33%) vs. 61 (43%) TPS cut‐off ≥ 1	82 (57%) vs. 80 (56%) CPS cut‐off ≥ 1% immune cell only	14.8 vs. 15.7 months	21 (15%) vs. 21 (15%)	10.1 vs. 8.6 months (HR 0.80; 95% CI 0.49–1.30)
OAK^74^	Phase III	Second line or later	Atezolizumab vs. docetaxel	112 (26%) vs. 110 (26%)	341 (80%) vs. 353 (83‐)	241 (57%) vs. 222 (51%) TPS cut‐off ≥ 1%	241 (57%) vs. 222 (51%) CPS cut‐off ≥ 1% immune cell only	21 months ‐ both groups	58 (14%) vs. 57 (13%)	13.8 vs. 9.6 months (HR 0.73; 95% CI 0.62–0.87; *P* = 0.0003)

Factors as histology, smoking status and PD–L1 expression are stated, as well as response rates and median overall survival (NA = not assessed; TPS = tumor proportion score; CPS = combined positive score).

Nivolumab monotherapy was granted FDA approval in pretreated patients with HNSCC and advanced LUSC following the respective CheckMate‐141 and CheckMate‐017 trials.^66^, ^67^ The tumor response to nivolumab was higher in LUSC patients than in HNSCC patients; however, overall the efficacy of nivolumab appeared relatively similar in HNSCC and LUSC. Recent data from the ATTRACTION‐3 study showed that nivolumab monotherapy was associated with a significant improvement in overall survival of 2.5 months compared to chemotherapy as second treatment of advanced or metastatic ESCC after progression on a platinum and fluoropyrimidime.^55^ In this study, which included mostly (96%) Asian patients, tumoral PD‐L1 expression could not predict treatment response. Based on these results, nivolumab is considered to be a promising second line treatment although differences are small. Also, the KEYNOTE‐180 and KEYNOTE‐181 study, which included PD‐L1 CPS > 1 ESCCs after failure of chemotherapy, observed statistically significant but small differences using PD‐1 inhibitor pembrolizumab as second line treatment.^56^, ^57^ However, patients with a PD‐L1 CPS score >10 had a median OS of 9.3 months in the pembrolizumab group compared to 7.8 months in the chemotherapy group. Therefore, in the United States, the Food and Drug Administration (FDA) has approved pembrolizumab monotherapy for patients with recurrent, locally advanced or metastatic ESCC expressing PD‐L1 (CPS ≥ 10), after 1 or more prior lines of systemic therapy in July 2019.

Besides the PD‐1 blockers pembrolizumab and nivolumab, humanized monoclonal antibodies have been developed that target PD‐L1 as well, namely durvalumab and atezolizumab. Blocking PD‐L1 prevents this ligand from binding to the PD‐1 receptor, aiming to reinstate the anti‐tumor response.^58^ The HAWK study investigated the safety and efficacy of durvalumab in HNSCC patients with progressive disease after platinum‐based chemotherapy with PD‐L1 expression of ≥ 25%. Results of this study showed an ORR of 16.2% with a median OS of 7.1 months.^68^ A similar study has been performed for NSCLC, namely the ATLANTIC study. This study investigated durvalumab alone, in advanced LUSC patients without previous exposure to any PD‐1 or PD‐L1 inhibitor. In cohorts 2 and 3, patients with LUSC were included. Cohort 2 consisted of patients with PD‐L1 expression of ≥ 25%, which is suitable to compare with the HAWK study on HNSCC. In this cohort, an ORR of 16.4% with a median OS of 10.9 months were observed.^69^ Very recently, data were published on the phase III EAGLE trial,^70^ in which patients with recurrent or metastatic HNSCC were randomized to receive durvalumab, durvalumab plus the anti‐CTLA4 ICI tremelimumab or SOC. No clinical benefit in OS was observed between durvalumab and SOC or durvalumab plus tremelimumab and SOC. However, durvalumab monotherapy did result in a higher response rate at 12‐ and 24‐months compared to SOC and the duration of response was also in favor of the ICI treatments compared to SOC. Results from the MYSTIC study, in which stage III and IV NSCLC patients were treated with durvalumab or durvalumab and tremelimumab vs. SOC, showed improved survival from double immunotherapy only in patients with blood Tumor Mutational Burden (TMB) > 20 per megabase.^71^

In HNSCC, only one clinical trial has been completed investigating safety and clinical activity of atezolizumab in patients with previously treated advanced HNSCC. An ORR of 22% was observed along with a median OS of 6.0 months.^72^ In NSCLC, atezolizumab was approved by the FDA following the phase II POPLAR and phase III OAK clinical trials, assessing the efficacy of this ICI vs. docetaxel in previously treated advanced NSCLC patients. Remarkably, in the case of atezolizumab, a higher ORR was observed in HNSCC than in LUSC; however, the duration of this response and the OS are significantly shorter. The higher ORR in HNSCC may be explained by the higher number of patients with profound PD‐L1 expression.^72^ To note, it is challenging to compare these studies due to the small patient cohort size (*n* = 32) in the study investigating atezolizumab in HNSCC vs. the larger patient cohorts in the NSCLC studies (*n* = 287 and *n* = 850, respectively).^72^, ^73^, ^74^ PD‐L1 blockers have, thus far, not been used in ESCC patients.

## Differences in immune landscapes linked to differences in PD1/PD‐L1 ICI response in HNSCC, LUSC and ESCC

As discussed, the overall efficacy of ICI targeting the PD‐1/PD‐L1 axis is higher in LUSC than in HNSCC and ESCC (see Tables 2 and 3). Several factors could contribute to this difference such as HPV‐infection, high mutational burden related to smoking and the presence of TLS,^75^, ^76^ for which heterogeneity is observed in immune cell infiltrate and IC expression (such as PD‐L1) in the TME.

Another factor that potentially could affect therapy response is a high mutational burden due to smoking. Rizvi *et al*. investigated as to whether the mutational landscape of NSCLC affects ICI treatment efficacy. This study demonstrated, in two independent cohorts, that a higher mutational burden resulted in improved response, duration of response and PFS, along with the observation that this improved efficacy correlated with smoking related gene signatures and higher neo‐antigen burden.^77^ Furthermore, a meta‐analysis performed by Kim *et al*. showed that ICI treatment prolonged OS and PFS in current and former smokers, however, did not improve survival in never smokers.^78^ Although most studies indicate an advantage in ICI efficacy in current and former smokers in NSCLC, this effect was not observed in the POPLAR and OAK studies investigating atezolizumab vs. docetaxel in NSCLC patients.^79^

For HNSCC, the link between smoking status, tumor mutational burden and response to ICI has not yet reached a consensus, with several studies reporting a more favorable HR in former smokers or high TMB tumors of ICI compared to SOC,^62^, ^80^, ^81^ and others reporting the opposite.^44^ The relationship might also be masked by the mixture of HPV+ and HPV− tumors, differences in TMB as well as differences in immune context and treatment response between those etiologies. The observation that a high TMB with a smoking‐related gene signature results in strong immunosuppressive effects and reduced levels of immune infiltrate, corresponds with the observation of decreased ICI efficacy in current or former smokers.^44^ Also, as mentioned, smoking is related to decreased expression of PD‐L1, which likely affects ICI therapy response as well.^82^, ^83^ Also in ESCC, which often display a high mutational burden, an immune cold and suppressive microenvironment is most prominent.^25^, ^26^

Performing digital spatial profiling on seven HNSCC tumor specimens (both HPV+ and HPV−), Kulasinghe *et al*. recently showed that while CD8^+^ T‐cell infiltration was not linked to response to ICI, other markers (CD44, CD45, CD4, CD68 and CD66B), more indicative of myeloid cells and Thelper cells, were linked with disease progression on ICI treatment.^84^

## Summarizing remarks

**Figure 1:**
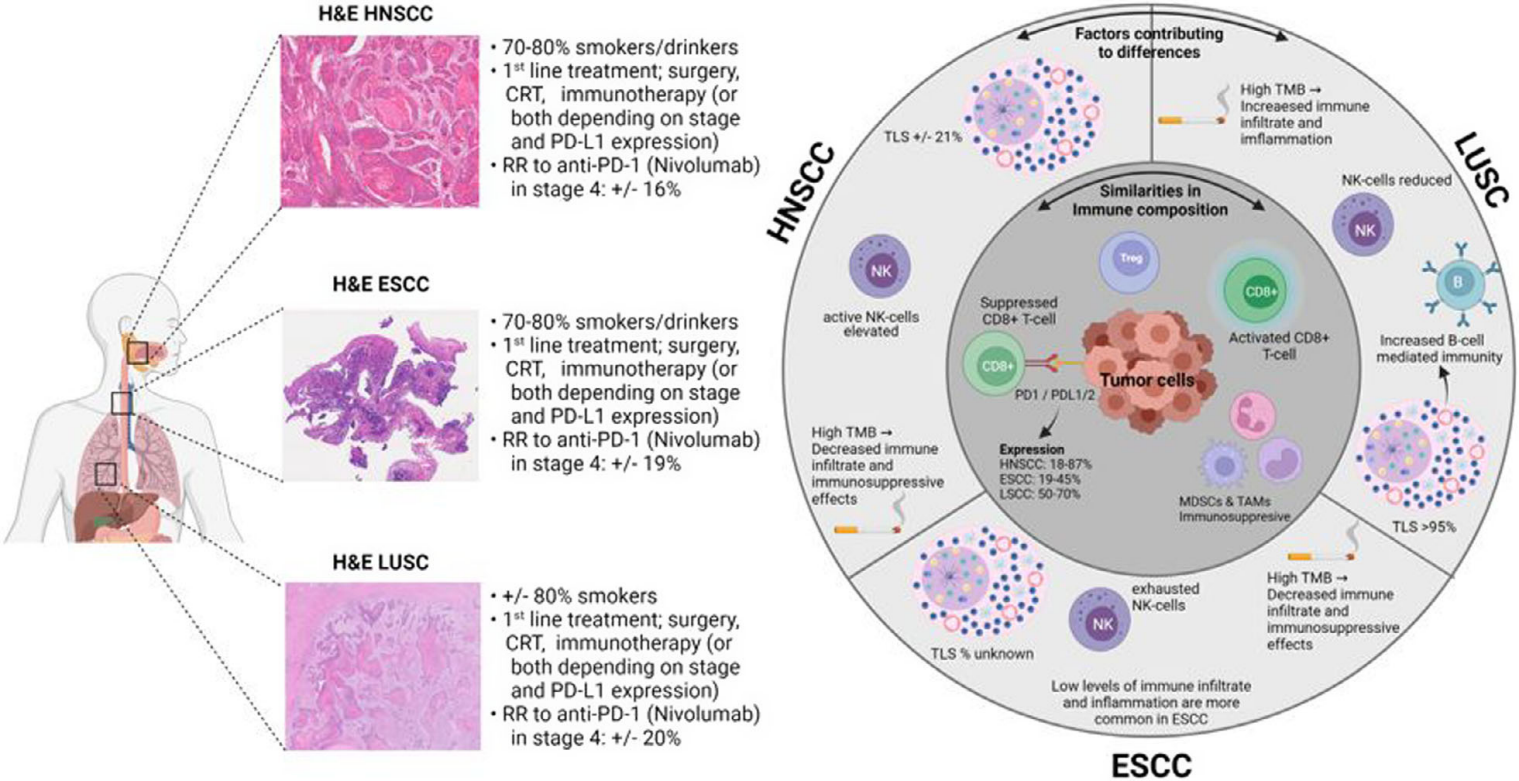
Schematic representation of comparison of HNSCC, ESCC and LUSC tumor microenvironment.

While ICI are mostly investigated in the context of tumors within distinct anatomical locations, there is increasing evidence that there are factors shared by SCC from different primary organs that might predict response to these treatments. SCC share more similarities among this histologic subtype than with tumors from a different histology in the same anatomical location.^3^ Many similarities are observed in the immune landscape of SCC; however, factors such as smoking and TLS‐abundance contribute to differences in the immune landscape of these tumors^44^, ^50^, ^76^, ^81^ (Figure 1). TLS are associated with increased anti‐tumor immunity and prognosis in LUSC^76^ and HNSCC^47^ and the role of high mutational burden due to a smoking history is unclear. In LUSC, a smoking‐related high mutational burden results in increased immune infiltrate, which either has an anti‐tumor activity or is suppressive, and increased expression of PD‐L1 is observed. This seems to result in higher response to ICI treatment in current or former smokers. The opposite is observed in HNSCC as smoking‐related high mutational burden appears to be associated with less immune infiltrate and strong immunosuppressive effects, as well as reduced levels of PD‐L1 expression. These observations most likely impede ICI therapy response. More recent research has pointed out distinct IS within SCC, which were discussed previously in this review (see Table 1).^50^ Two IS were observed which had higher expression of gene signatures related to inflammation and T cells with increased cytolytic activity than the other subtypes (IS4 and IS5) and these are hypothesized to benefit the most from ICI treatment.^50^ The distribution of IS4 and IS5 in LUSC, HNSCC and ESCC may indicate a predictive factor for response to ICI. Together, a total of 40% of LUSC encompass these two subtypes, which was 29% for HNSCC and 11% of ESCC.^50^ The fact that this percentage is higher in LUSC may explain why ICI tend to have a higher efficacy than that in HNSCC and ESCC (as observed by HRs of OS of ICI vs. SOC; see Tables 2 and 3). In HPV− HNSCC, tumors can be located at different anatomical locations within the head and neck area, which may also influence their interaction with immune cells.^20^ Looking at the different signatures, high TGF‐β presence seems to be a dominant feature in HNSCC, as well as ESCC, and TGF‐β has been linked to reduced T‐cell infiltration into the tumor and reduced efficacy of ICI therapy.^85^, ^86^, ^87^ The less immune inflamed IS may be eligible to treatments that convert a cold immune landscape into a hot immune landscape, such as radiotherapy and chemotherapy, or novel treatment options as oncolytic viruses, cancer vaccines or combinations with TGF‐β inhibitors.

Altogether, the available literature indicates the many similarities among SCC arising in distinct anatomical locations; however, the differences observed in immune landscapes indicate that, in the future, ICI treatments (and other immunotherapeutics) are probably best chosen based on immune cell infiltrate, IC expression and other immune related, and potentially also tumor molecular factors, tending towards personalized therapy.

## Conflicts of interest

The authors declare no conflicts of interest.

## Author contribution

**Maurice van Duijvenvoorde:** Methodology; Writing – original draft. **Sarah Derks:** Methodology; Writing – review & editing. **Idris Bahce:** Writing – review & editing. **CR Leemans:** Writing – review & editing. **Rieneke van de Ven:** Conceptualization; Methodology; Supervision; Writing – original draft; Writing – review & editing. **Marieke F Fransen:** Conceptualization; Methodology; Supervision; Writing – original draft; Writing – review & editing.
